# Joint Experimental and Computational Characterization of Sum-Frequency Generation between a Continuous Wave Laser and an Ultrafast Frequency Comb Laser for Tunable Laser Development

**DOI:** 10.1021/acsphotonics.4c01783

**Published:** 2025-01-29

**Authors:** Jie Zhan, Nicholas D. Cooper, Melanie A. R. Reber

**Affiliations:** Department of Chemistry, University of Georgia, Athens, Georgia 30602, United States

**Keywords:** nonlinear optics, frequency comb lasers, nonlinear frequency conversion, three-wave mixing, sum-frequency generation, ultrafast tunable laser, ultrafast frequency comb laser, SNLO simulation

## Abstract

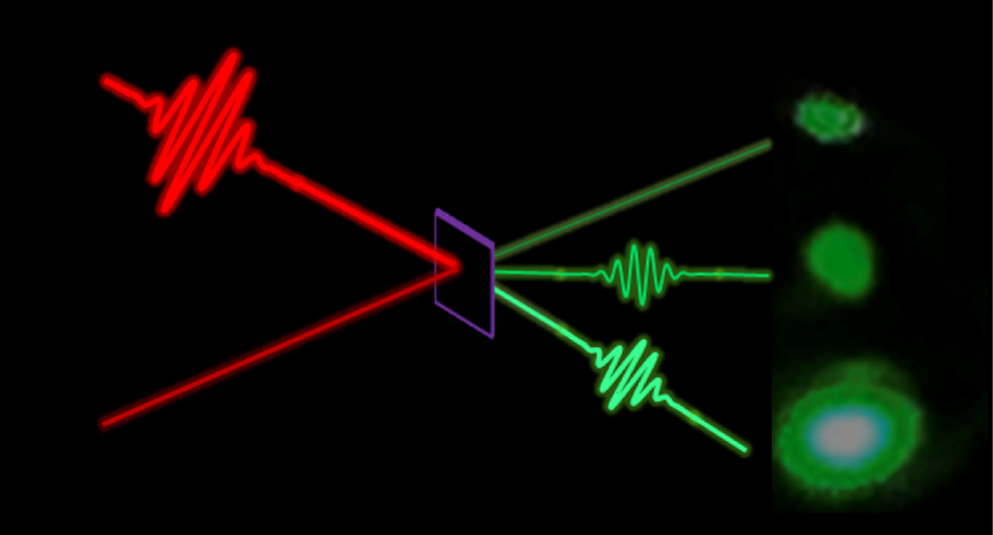

Ultrafast optical frequency combs allow for both high spectral and temporal resolution in molecular spectroscopy and have become a powerful tool in many areas of chemistry and physics. Ultrafast lasers and frequency combs generated from ultrafast mode-locked lasers often need to be converted to other wavelengths. Commonly used wavelength conversions are optical parametric oscillators, which require an external optical cavity, and supercontinuum generation combined with optical parametric amplifiers. Whether commercial or home-built, these systems are complex and costly. Here, we investigate an alternative, simple, and easy-to-implement approach to tunable frequency comb ultrafast lasers enabled by new continuous-wave laser technology. Sum-frequency generation between an Nd:YAG continuous-wave laser and a Yb:fiber femtosecond frequency comb in a beta-barium borate (BBO) crystal is explored. The resulting sum-frequency beam is a pulsed frequency comb with the same repetition rate as the Yb:fiber source. SNLO simulation software is used to simulate the results and provide benchmarks for designing future systems to achieve wavelength conversion and tunability in otherwise difficult-to-reach spectral regions.

## Introduction

Combining high temporal resolution and wide spectral coverage, ultrafast optical spectroscopy is used in a wide range of fields including chemical dynamics^1,2^ photoinduced dynamic processes,^3,4^ and materials characterization.^5^ Ultrafast lasers are also used to generate optical frequency combs, the narrow spectral line widths provide ultrahigh precision in both time and spectral resolution.^6−8^ Frequency combs have found application in precision metrology and molecular spectroscopy.^6,9−11^ Mode-locked lasers, including Ti:sapphire lasers and fiber lasers, are the most widely used sources for ultrafast pulse generation, including frequency comb lasers. To cover the spectral regions of interest to molecular spectroscopy and other applications, it is generally necessary to frequency convert the light external to the laser. Common setups for creating tunable radiation from a mode-locked ultrafast laser are optical parametric oscillators (OPO) or optical parametric amplifiers (OPA) combined with supercontinuum generation.

Ultrafast OPOs have been built for a range of wavelength regions, pulse durations, and tunability bandwidths. They all utilize an optical cavity with an intracavity nonlinear crystal and are pumped with mode-locked laser.^12−14^ OPOs involve coupling the incoming laser to an external optical cavity, which contributes to the complexity and environmental demands of an instrument. Active feedback and control are necessary for high-performance OPOs and involved PID controllers and feedback mechanisms.^15,16^ Frequency tunability of ultrafast frequency comb lasers also use OPOs pumped by mode-locked lasers.^16,17^

Ultrafast OPAs use a high-power pulsed laser to amplify a seed laser in a nonlinear crystal. The seed is often a tunable source made through supercontinuum generation.^18,19^ OPAs are commonly seeded with kHz repetition rate lasers, which can undergo supercontinuum generation in a range of materials, such as sapphire and calcium fluoride. Lower repetition rate lasers (100 MHz or less) have lower peak powers for the same average power so supercontinuum generation is usually done in a nonlinear fiber, which can have stability problems. This limitation on supercontinuum materials ultimately presents a limitation in using them for high-repetition-rate tunable lasers, even in an OPA configuration. Additionally, OPAs require the overlap in both space and time of the pump laser and the seed laser, which adds to the experimental complexity.^20,21^

The idea of generating frequency-tunable pulses to get around the challenges of OPOs and OPAs has been a topic of considerable interest that most often focuses on supercontinuum generation.^22^ However, taking advantage of the developments in stable, tunable, continuous wave (CW) sources coming on the market, several groups revisited the possibility of using difference frequency generation (DFG) and sum frequency generation (SFG) to make tunable ultrafast light without an OPO configuration. There are currently limited studies, three to the best of our knowledge, aimed at achieving frequency conversion^23−25^ with this route; however, the studies were mostly done in the picosecond or subpicosecond regime without providing calculations to enable the design of other systems, such as with commercially available lasers or at other wavelengths and crystals. Two of the three used PPLN crystals in a quasi-phase-matching regime, which is more limited in the possible frequency regions reachable. We investigate three-wave mixing between CW and ultrafast laser in a nonlinear crystal and model it using theory, to lay the foundation for further implementation for a range of wavelength regions.

Specifically, three-wave mixing is achieved through sum frequency generation in a BBO crystal between an ultrafast Yb:fiber frequency comb centered at 1055 nm and an Nd:YAG CW laser centered at 1064 nm. The ultrafast Yb:fiber laser is an optical frequency comb.^26,27^ Each optical comb tooth has a frequency, *ν*_*n*_(*Yb*:*fiber*), and will undergo sum-frequency generation with the single-frequency Nd:YAG laser:
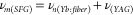
1

The resulting SFG light will be a frequency comb with the frequency of each comb tooth, *ν*_*m*_(*SFG*), equal to the sum frequency of a single Yb:fiber comb tooth with the CW laser. This is analogous to that seen in difference frequency generation between two frequency combs.^28^

To the best of our knowledge, sum-frequency mixing between CW and femtosecond laser has been reported only once before by Salhi et al.,^25^ who demonstrated SFG in a beta barium borate (BBO) crystal between a femtosecond Ti:sapphire laser with 130 fs pulse duration and center wavelength of 797 nm, and a GaInAsP/InP CW laser, with emission wavelength at 1551 nm. Their resulting SFG beam had a center wavelength of 527 nm, a power of 10 pW, and a conversion efficiency of 7 × 10^–10^. A systematic study, optimization of conversion efficiency, and comparison to computational predictions are still required to be able to use this method for a tunable laser design. Here we expand upon that first observation by systematically characterizing the SFG between a CW laser and ultrafast frequency comb laser and comparing the results to calculations made with SNLO, a software to perform detailed simulations of nonlinear mixing processes in nonlinear crystals.^29^

## Experimental Setup

**Figure 1:**
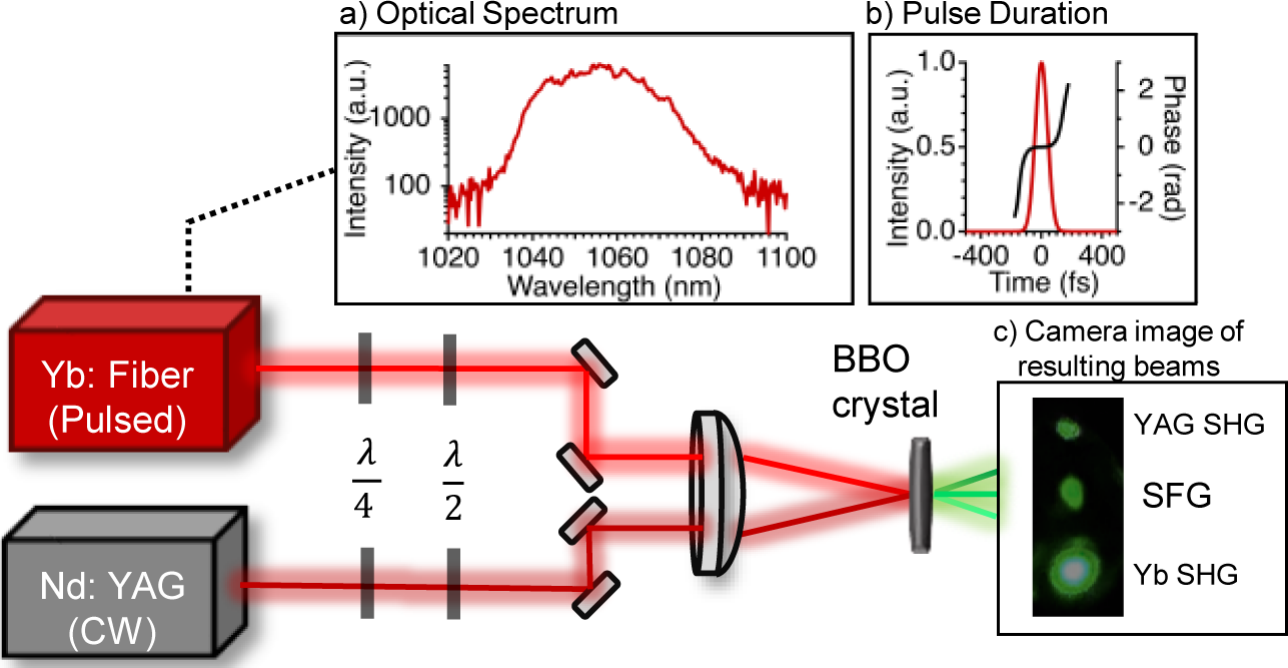
Experimental setup scheme. Inset: (a) spectrum of Yb:fiber comb and (b) pulse profile from FROG measurement, (c) a photo of the YAG SHG, Yb SHG, and SFG beam (captured by iPhone 14 Pro).

The experimental setup is shown in Figure 1. The ultrafast laser is a home-built 85 MHz Yb:fiber laser frequency comb and Yb:fiber photonic crystal chirped-pulse amplifier^11,27^ with a center wavelength of 1055 nm, 20 nm fwhm (Figure 1a, the intensity of the spectrum is in log scale) and a maximum power of 9 W. An intracavity bulk EOM and μm-scale piezo-electric adjustments stabilize the comb. It has a 97 fs pulse duration (Figure 1b) measured by the frequency-resolved optical gating technique (Swamp Optics GRENOUILLE). In this work, frequency comb powers from 200 to 500 mW are used in order to stay about an order of magnitude lower than the damage threshold of the nonlinear crystal at the small beam sizes employed. The CW source is a stable nonplanar ring oscillator Nd:YAG laser (Coherent Mephisto) with a center wavelength of 1064 nm, 3 kHz line width, and up to 500 mW output power, of which 20 to 110 mW is used, delivered via optical fiber to the ultrafast laser table. The beam sizes of the two sources are made to match by Keplerian telescopes, and waveplates to align the polarization of both beams, and then both are focused by a 50 mm plano-convex lens to a 5 × 5 × 1.0 mm BBO crystal with a Type I phase matching scheme. The ABCD matrix method is used to calculate the beam size and radius of curvature of both lasers at the BBO crystal, based upon beam profile measurements made with the knife-edge method.

Sum-frequency generation between the CW laser and the frequency comb source is achieved under a noncollinear regime, with a relative angle of 11° and a 5.5° angle to the crystal normal. This is analogous to an autocorrelation geometry where the resulting CW (YAG) SHG, pulsed (Yb) SHG, and the SFG beam are spatially separated. A photograph of the three generated beams is shown in Figure 1. The incident angle of the crystal can be tuned to optimize the SHG of either the YAG or Yb lasers or the SFG between the two. The image shows an incident angle where all three were visible with the camera. Generated beam power was optimized prior to taking data. The SFG and SHG beams are characterized by a grating spectrometer (ASEQ LR1), a power meter (Thorlabs S130C), and an RF spectrum analyzer (Rigol DSA815).

## Results and Discussions

**Figure 2:**
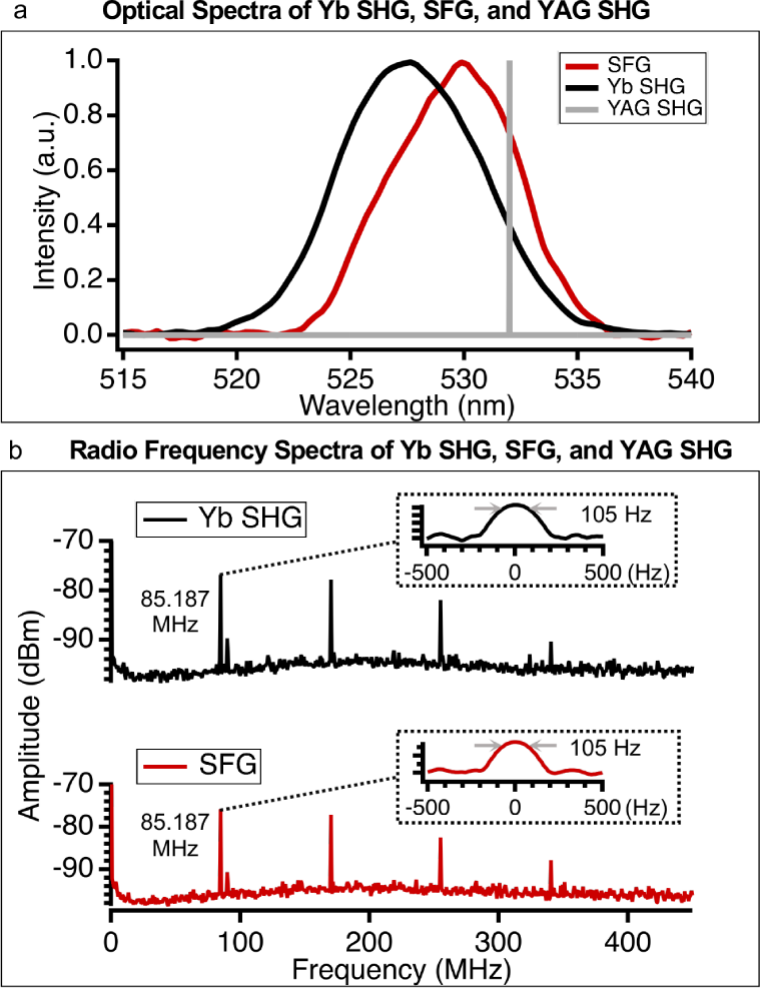
(a) Optical spectra of mixed SFG and ultrafast SHG (experimental measurements), CW SHG (drawn) on a linear scale for clarity; (b) RF spectra of mixed SFG and Yb:fiber SHG. The repetition rate of mixed SFG and Yb:fiber SHG is 85 MHz. 170, 255, and 340 MHz are harmonics of the repetition rate of Yb:fiber SHG and SFG pulses (RBW = 1 MHz). The signal at 90.5 MHz is the local campus radio station. Insets are more detail of the repetition rate of Yb:fiber SHG and SFG signals (measurement RBW = 10 Hz); both widths are therefore limited by the instrument bandwidth.

Figure 2a presents the optical spectra of the Yb:fiber SHG and SFG beam. The Yb:fiber laser is a frequency comb, so for each comb tooth within the phase-matching bandwidth it will follow eq 1. Individual comb teeth cannot be resolved with the grating spectrometer. The SFG beam has a center wavelength of 530 nm, while the CW SHG is at 532 nm, and pulsed SHG is at 528 nm. The center wavelength of the SFG beam agrees with the theoretically expected value. For the ultrafast Yb:fiber SHG process with an interaction length with BBO of 1 mm, the calculated fwhm phase-matching bandwidth^30^ is 21.1 nm, while the fwhm of our Yb:fiber source is about 20 nm. With the phase-matching bandwidth slightly larger than the fwhm of the Yb:fiber source, the SHG bandwidth should be 10 nm. For the SFG process, the bandwidth of the Yb:fiber is much greater than that of the CW source, so the predicted SFG fwhm is also 10 nm. The experimentally measured optical fwhm of the SFG signal is 7 nm, and the fwhm of the Yb:fiber SHG signal is 8 nm. The reduced bandwidth of the SHG and SFG processes may also be due to the spatial walk-off effect by the femtosecond source.

The walk-off caused by group velocity mismatch is compared with the phase mismatch to see which effect likely dominates the SFG conversion efficiency. The phase mismatch Δ*k* is calculated based on the refractive index provided by SNLO (−3.468/mm) and gives a coherent length *L_coh_* = 2π/Δ*k* = 1.811 mm.^30^ The group velocity mismatch is Δβ*′* =1/ν_*SFG*_ – 1/ν_Yb_ = 8.66 × 10^–11^ s/m. For the τ = 97 fs pulse duration of Yb:fiber laser, the walk-off length caused by group velocity mismatch is therefore *L*_*g*_ = τ/Δβ*′* = 1.12 mm. Since *L*_*g*_ < *L*_*coh*_, the group velocity mismatch will dominate and cause additional conversion efficiency decrease.

To confirm that the SFG beam is pulsed, a photodetector captured the ultrafast SHG and SFG signal and recorded it with the RF spectrum analyzer to get the repetition rate of the beams, shown in Figure 2b. The SFG beam gives an 85 MHz repetition rate, which agrees with the Yb:fiber source. The 3 dB bandwidth for both Yb SHG and SFG signals is 105 Hz, consistent with the lower limit of the spectrum analyzer and confirming that the generated SFG beam has the same pulse repetition rate as the SHG beam.

**Figure 3:**
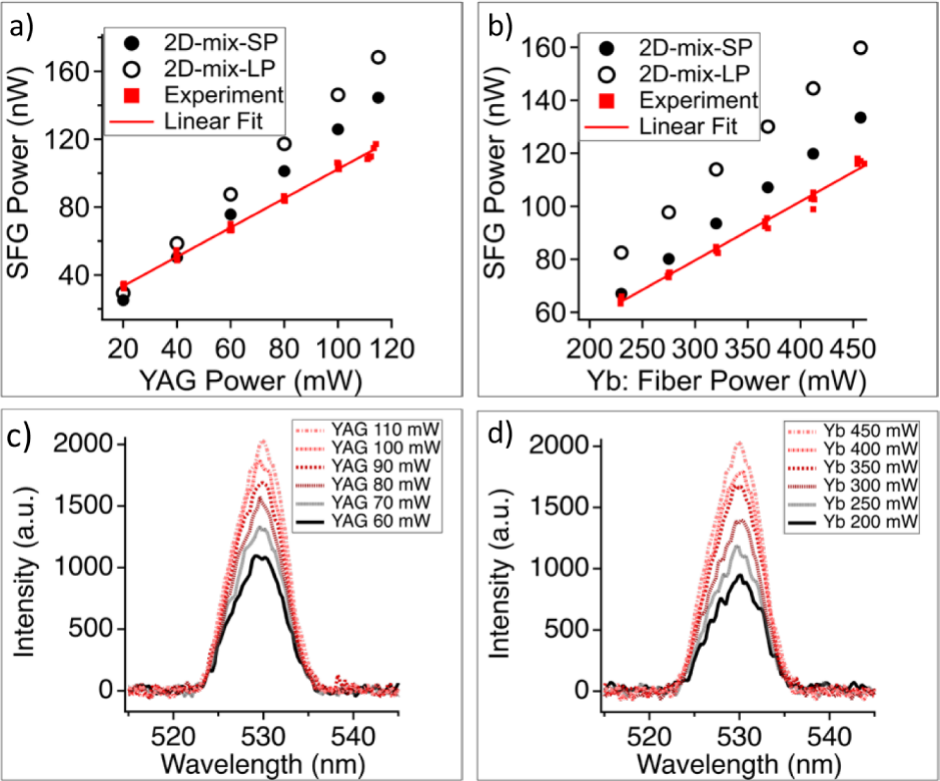
(a) SFG power of simulation and experimental result with 470 mW ultrafast Yb:fiber power; (b) SFG power of simulation and experimental result when power of YAG (CW) of 110 mW; (c) spectra of SFG beam with a Yb:fiber(ultrafast) power of 470 mW; (d) spectra of SFG beam with CW Nd:YAG power of 110 mW.

Figure 3a plots the generated SFG power with respect to the CW Nd:YAG laser power while the ultrafast Yb:fiber laser was held at 470 mW and Figure 3b plots the generated SFG power while varying the ultrafast Yb:fiber laser power and keeping the CW laser at 110 mW. The data was taken five different times, all plotted on the graph, and least-squares fit to a line (slope and intercept). Figure 3c,d shows the optical spectra of the SFG light for the same set of powers. As evident from the plot, the spectra of the SFG do not change significantly as the power of the CW laser (c) or ultrafast laser (d) is increased. The SFG power depends linearly on the CW source power and also linearly on the ultrafast source power, which is consistent with an overall second-order process that is first-order with each laser source. The slope of each linear fit between SFG and CW power, as well as between SFG and Yb:fiber power, represents the conversion efficiency of SFG relative to these two sources. The slope efficiency for SFG is η*_SFG/CW_* = *P_SFG_*/*P_CW_* = 8.6 (0.1) × 10^–7^ with respect to CW source power and η_*SFG/pulsed*_ = *P_SFG_/P_pulsed_* = 2.22 (0.05) × 10^–7^ with respect to the ultrafast source power. For comparison, the experimental SHG conversion efficiency has a slope of 0.059 (0.002) for the ultrafast Yb:fiber laser and 7.5 (0.2) × 10^–7^ for the CW YAG laser. The much lower CW conversion efficiency is due to the significantly lower effective peak power in the three-wave mixing process. Since the power of SFG is proportional to both pump powers, SFG achieved a higher conversion efficiency compared to CW SHG because of the higher peak power of the ultrafast laser. Conversely, SFG conversion efficiency with respect to the ultrafast laser is being pushed lower because of the low peak power of the CW laser. The normalized conversion efficiency considering both CW and ultrafast laser, *P_SFG_*/(*P_CW_* × *P*_*pulsed*_)^31,32^ is 2.0 (0.1) μW/W^2^. Note that the CW power used above represents the energy of CW laser per second, including the portion that does not interact with the ultrafast source, specifically between each Yb:fiber laser pulse. If we exclude the noninteracting portion in the energy calculation, the SFG conversion efficiency with respect to the CW laser is 10.4%. The photon conversion efficiency analyses supports the idea that the low peak power of the CW source is the major limiting factor for the SFG conversion efficiency, as expected.

To complement the experimental results, we performed simulations using SNLO^29^ to predict conversion efficiencies. The standard procedures available in SNLO do not address the scenario of a pulsed laser, specifically an ultrafast laser, mixing with a CW laser. We therefore compared results using both the long pulse (2D-mix-LP) and short pulse (2D-mix-SP) programs with the experimental results. The two-dimensional long-pulse mixing (2D-mix-LP) is a model for nonlinear single-pass mixing for long pulse durations up to CW lasers. It includes the effects of Gaussian spatial profiles, birefringent walk-off, and diffraction but ignores group velocity effects.

**Table 1:** Parameters for SNLO Simulation

Parameter	CW	Pulsed	SFG
Wavelength (nm)	1064(o)	1055(o)	529.7(e)
Phase Velocity	c/1.654241	c/1.654408	c/1.654364
Group Velocity	c/1.67390	c/1.67410	c/1.70008
GDD (fs^2^/mm)	44.014	44.929	129.094
Walk-off Angle (mrad)	0	0	55.9
*d*_eff_ (pm/V)	1.994		
Δ*k* (1/mm)	–3.468		

The two-dimensional short-pulse mixing (2D-mix-SP) is a model for ps and fs pulses that incorporates group velocity effects. It also includes dispersion and diffraction for Gaussian beams. The upper limit for pulse duration in 2D-mix-SP is 100 ps, so the CW laser “pulse duration” is set to the upper limit of 100 ps to use this model. In both 2D-mix-LP and 2D-mix-SO, the fitting parameters in Table 1 are retrieved from SNLO under the designated phase matching condition. Other input parameters including pulse duration, power, and Gaussian beam parameters are experimentally measured or calculated using the ABCD matrix method. Since the peak power of a Gaussian pulse is about 0.94 times the pulse energy divided by pulse duration, we calculated the simulation’s pulse energy based on the CW power and used it to match the experimental conditions.

The results given by the 2D-mix-LP are slightly larger in SFG power compared to the 2D-mix-SP in Figure 3a,b. The lack of group velocity mismatch taken into account by the model likely accounts for this, as discussed earlier. Therefore, the long-pulse simulated SFG power is expected to be larger than the short-pulse simulated power. However, since the interactive length for SFG is only 1 mm, the group velocity mismatch error is only a relatively small difference between the two simulation types as seen in Figure 3.

The experimental results are systematically lower in conversion efficiency compared to the 2D-mix-SP simulation. This is likely a result of the noncollinear geometry used in the experiment as compared to the collinear geometry of the simulation. In a noncollinear geometry there is an additional phase mismatch resulting from the finite crossing angle of the beams as they focus on the crystal. In addition, although SNLO takes beam focusing into account, there are some small errors in the experimental measurement and subsequent ABCD matrix calculation of beam parameters. According to our ABCD matrix calculation and experimental beam diameter measurement,^33^ the Rayleigh range *z*_*R*_ of the source is 0.59 mm, which is shorter than the interaction length of 1 mm. The optimal focus and crystal position were found by experimentally optimizing the SFG output power, which leaves a small uncertainty in the beam parameters to put into the SNLO simulation. Therefore, we used the ultrafast Yb:fiber SHG experimental result, which the simulation is designed to work for, and slightly adjusted the beam diameter and radius of curvature to get the SHG data to match the simulation output. We then used those adjusted parameters as the beam input parameters in the SFG simulation.

## Conclusion and Future Perspectives

This experiment investigated sum-frequency generation between a CW laser and an ultrafast frequency comb laser. The resulting SFG beam has the repetition rate of the ultrafast laser and the corresponding broad spectra. The total output powers achieved with these input power are low, but sufficient for a cavity-enhanced absorption experiment and other experiments that do not require high power. The SNLO simulation of SFG power agrees with our observation, which will be used to optimize the conversion efficiency in upcoming research. This experiment provides the foundation to use this concept of mixed ultrafast-CW nonlinear frequency generation. By utilizing a commercial tunable CW laser or sets of CW lasers, such as quantum-cascade lasers, external-cavity diode lasers (ECDL), or CW fiber lasers, paired with appropriate amplifiers if needed, one could achieve frequency tunability by only changing the phase matching angle of BBO crystal in a three-wave mixing process, without implementing cavities or delay stages. The successful realization of SFG suggests that its inverse process, DFG, could also be achieved. With DFG, tunable mid-IR frequencies are accessible. A visible or NIR supercontinuum frequency comb^34^ could be used as the ultrafast source to attain a mid-IR supercontinuum frequency comb with this setup. Based on currently available commercial CW lasers and a stock nonlinear crystal, we can use SNLO to assist design a reasonable setup with a predicted tunable wavelength range and output power after frequency conversion. For example, with 15 W from a commercially available 1550 nm, narrow-line width CW Er-doped fiber laser with amplifier combined with 9 W of our Yb:fiber ultrafast laser at 1060 nm, DFG in a commercially stocked, 2 cm long KTP crystal gives 335 mW of ultrafast light at 3.3 μm. At these wavelengths, the walk-off in the KTP crystal is suitable small to allow for use of a longer crystal for higher power. This is sufficient power for many absorption experiments requiring either ultrafast or frequency combs or both.

With the high powers now easily achievable in ultrafast Yb:fiber lasers,^11^ ample power for the ultrafast and CW lasers is available to compensate for the lower conversion efficiency and lack of cavity of OPO. Our work has demonstrated the viability of three-wave mixing between a frequency comb and a CW laser, providing support for the core idea and design criteria behind the simplified designs of tunable ultrafast frequency comb lasers.

## Notes

Jie Zhan; Nicholas D. Cooper; Melanie A. R. Reber. Three-Wave Mixing between Continuous-Wave and Ultrafast Lasers. 2024, arXiv:2405.04424v1. arXiv. https://arxiv.org/abs/2405.04424v1 (accessed May 7, 2024).

## References

[ref1] Nibbering E. T.; Fidder H.; Pines E. ULTRAFAST CHEMISTRY: Using Time-Resolved Vibrational Spectroscopy for Interrogation of Structural Dynamics. Annu. Rev. Phys. Chem. 2005, 56, 337–367. 10.1146/annurev.physchem.56.092503.141314. 15796704

[ref2] Butler J. M.; George M. W.; Schoonover J. R.; Dattelbaum D. M.; Meyer T. J. Application of transient infrared and near infrared spectroscopy to transition metal complex excited states and intermediates. Coord. Chem. Rev. 2007, 251, 492–514. 10.1016/j.ccr.2006.12.002.

[ref3] Maiuri M.; Garavelli M.; Cerullo G. Ultrafast Spectroscopy: State of the Art and Open Challenges. J. Am. Chem. Soc. 2020, 142, 3–15. 10.1021/jacs.9b10533. 31800225

[ref4] Di Donato M.; Groot M. L. Ultrafast infrared spectroscopy in photosynthesis. Biochim. Biophys. Acta, Bioenerg. 2015, 1847, 2–11. 10.1016/j.bbabio.2014.06.006. 24973600

[ref5] Munson K. T.; Kennehan E. R.; Asbury J. B. Structural origins of the electronic properties of materials via time-resolved infrared spectroscopy. J. Mater. Chem. C 2019, 7, 5889–5909. 10.1039/C9TC01348B.

[ref6] Cingöz A.; Yost D. C.; Allison T. K.; Ruehl A.; Fermann M. E.; Hartl I.; Ye J. Direct Frequency Comb Spectroscopy in the Extreme Ultraviolet. Nature 2012, 482, 68–71. 10.1038/nature10711. 22297971

[ref7] Udem T.; Holzwarth R.; Hänsch T. W. Optical Frequency Metrology. Nature 2002, 416, 233–237. 10.1038/416233a. 11894107

[ref8] Picqué N.; Hänsch T. W. Frequency Comb Spectroscopy. Nat. Photonics 2019, 13, 146–157. 10.1038/s41566-018-0347-5.

[ref9] Keilmann F.; Amarie S. Mid-Infrared Frequency Comb Spanning an Octave Based on an Er Fiber Laser and Difference-Frequency Generation. J. Infrared, Millimeter, Terahertz Waves 2012, 33, 479–484. 10.1007/s10762-012-9894-x.

[ref10] Bartels A.; Heinecke D.; Diddams S. A. 10-GHz Self-Referenced Optical Frequency Comb. Science 2009, 326, 681–681. 10.1126/science.1179112. 19900924

[ref11] Li X.; Reber M. A. R.; Corder C.; Chen Y.; Zhao P.; Allison T. K. High-power Ultrafast Yb: Fiber Laser Frequency Combs Using Commercially Available Components and Basic Fiber Tools. Rev. Sci. Instrum. 2016, 87, 093114 10.1063/1.4962867. 27782582

[ref12] Geesmann F. J.; Mevert R.; Zuber D.; Morgner U. Rapidly Tunable Femtosecond Near-UV Pulses from a Non-Collinear Optical Parametric Oscillator. Opt. Express 2023, 31, 27296–27303. 10.1364/OE.498170. 37710808

[ref13] Mevert R.; Binhammer Y.; Dietrich C. M.; Beichert L.; de Andrade J. R. C.; Binhammer T.; Fan J.; Morgner U. Widely Tunable, High-Power, Femtosecond Noncollinear Optical Parametric Oscillator in the Visible Spectral Range. Photonics Res. 2021, 9, 1715–1718. 10.1364/PRJ.426107.

[ref14] Deckert T.; Vanderhaegen A.; Brida D. Sub-8-fs Pulses in the Visible to Near-Infrared by a Degenerate Optical Parametric Amplifier. Opt. Lett. 2023, 48, 4496–4499. 10.1364/OL.498291. 37656537

[ref15] Reid D. T.; Sun J.; Lamour T. P.; Ferreiro T. I. Advances in Ultrafast Optical Parametric Oscillators. Laser Phys. Lett. 2011, 8, 8 10.1002/lapl.201010085.

[ref16] Kobayashi Y.; Torizuka K.; Marandi A.; Byer R. L.; McCracken R. A.; Zhang Z.; Reid D. T. Femtosecond Optical Parametric Oscillator Frequency Combs. J. Opt. 2015, 17, 094010 10.1088/2040-8978/17/9/094010.

[ref17] Chen Y.; Silfies M. C.; Kowzan G.; Bautista J. M.; Allison T. K. Tunable Visible Frequency Combs from a Yb-Fiber-Laser-Pumped Optical Parametric Oscillator. Appl. Phys. B: Lasers Opt. 2019, 125, 81 10.1007/s00340-019-7191-2.

[ref18] Dalla-Barba G.; Jargot G.; Lassonde P.; Tóth S.; Haddad E.; Boschini F.; Delagnes J.-C.; Leblanc A.; Ibrahim H.; Cormier E. Mid-Infrared Frequency Domain Optical Parametric Amplifier. Opt. Express 2023, 31, 14954–14964. 10.1364/OE.487813. 37157348

[ref19] Xu D.; Zhang J.; He Y.; Wang Y.; Yao J.; Guo Y.; Yan C.; Tang L.; Li J.; Zhong K.; Wu Y.; Yao J. High-Energy, Tunable, Long-Wave Mid-Infrared Optical Parametric Oscillator Based on BaGa4Se7 Crystal. Opt. Lett. 2020, 45, 5287–5290. 10.1364/OL.401956. 32932513

[ref20] Brida D.; Manzoni C.; Cirmi G.; Marangoni M.; Bonora S.; Villoresi P.; De Silvestri S.; Cerullo G. Few-Optical-Cycle Pulses Tunable from the Visible to the Mid-Infrared by Optical Parametric Amplifiers. J. Opt. 2010, 12, 013001 10.1088/2040-8978/12/1/013001.

[ref21] Manzoni C.; Cerullo G. Design Criteria for Ultrafast Optical Parametric Amplifiers. J. Opt. 2016, 18, 103501 10.1088/2040-8978/18/10/103501.

[ref22] Wu T.-H.; Ledezma L.; Fredrick C.; Sekhar P.; Sekine R.; Guo Q.; Briggs R. M.; Marandi A.; Diddams S. A. Visible-to-ultraviolet frequency comb generation in lithium niobate nanophotonic waveguides. Nat. Photonics 2024, 18, 218–223. 10.1038/s41566-023-01364-0.

[ref23] Zhao J.; Chen Y.; Ouyang D.; Liu M.; Li C.; Wu X.; Xiong X.; Mo L.; Wang M.; Liu X. Over 3.8 W, 3.4 μm picosecond mid-infrared parametric conversion based on a simplified one-to-many scheme. Opt. Express 2024, 32, 8364–8378. 10.1364/OE.516265. 38439493

[ref24] Krzempek K.; Sobon G.; Sotor J.; Abramski K. A dual-wavelength amplifier that enables the simultaneous chirped-pulse amplification of femtosecond 1562 nm pulses and continuous wave 1064 nm radiation for applications in difference frequency generation. Laser Phys. Lett. 2016, 13, 105107 10.1088/1612-2011/13/10/105107.

[ref25] Salhi M.; Bieler M.; Koch M. Nonlinear Mixing between the Emission of a cw Laser and a Femtosecond Laser. Opt. Commun. 2007, 274, 198–200. 10.1016/j.optcom.2007.01.050.

[ref26] Reber M. A. R.; Chen Y.; Allison T. K. Cavity-Enhanced Ultrafast Spectroscopy: Ultrafast Meets Ultrasensitive. Optica 2016, 3, 311–317. 10.1364/OPTICA.3.000311.

[ref27] Cooper N. D. Construction of a Yb:fiber Frequency Comb for Ultrafast Gas Phase Spectroscopy. Ph.D. thesis, University of Georgia, 2024.

[ref28] Schliesser A.; Picqué N.; Hänsch T. W. Mid-Infrared Frequency Combs. Nat. Photonics 2012, 6, 440–449. 10.1038/nphoton.2012.142.

[ref29] AS Photonics. SNLO version 79. AS Photonics: Albuquerque, NM, 2024; https://as-photonics.com/products/snlo/.

[ref30] Saleh B. E. A.; Teich M. C. Nat. Photonics; John Wiley & Sons, 2019; pp. 1032–1033.

[ref31] Nishikawa T.; Ozawa A.; Nishida Y.; Asobe M.; Hong F.-L.; Hänsch T. W. Efficient 494 mW sum-frequency generation of sodium resonance radiation at 589 nm by using a periodically poled Zn: LiNbO 3 ridge waveguide. Opt. Express 2009, 17, 17792–17800. 10.1364/OE.17.017792. 19907566

[ref32] Mimoun E.; Sarlo L. D.; Zondy J.-J.; Dalibard J.; Gerbier F. Sum-frequency generation of 589 nm light with near-unit efficiency. Opt. Express 2008, 16, 18684–18691. 10.1364/OE.16.018684. 19581954

[ref33] Suzaki Y.; Tachibana A. Measurement of the μm Sized Radius of Gaussian Laser Beam Using the Scanning Knife-Edge. Appl. Opt. 1975, 14, 2809–2810. 10.1364/AO.14.002809. 20155108

[ref34] Ruehl A.; Martin M. J.; Cossel K. C.; Chen L.; McKay H.; Thomas B.; Benko C.; Dong L.; Dudley J. M.; Fermann M. E. Ultrabroadband Coherent Supercontinuum Frequency Comb. Phys. Rev. A 2011, 84, 011806 10.1103/PhysRevA.84.011806.

